# Chylothorax as Initial Presentation of Follicular Lymphoma: A Case Report and Literature Search

**DOI:** 10.1155/2024/7985228

**Published:** 2024-06-06

**Authors:** Gi Eun Kim, Yousif Khaled, Saleh Mahmoud, Amin Ur Rehman

**Affiliations:** Department of Internal Medicine, Hamad General Hospital, Hamad Medical Corporation, Doha, Qatar

## Abstract

Chylothorax is accumulation of chyle in pleural space. Causes include traumatic, such as after esophagectomy, and nontraumatic, most commonly malignancy. Lymphoma usually presents as asymptomatic lymphadenopathy, and chylothorax tends to occur late in disease course. Chylothorax as initial presentation of lymphoma is rare with only case reports. We present a case of 43-year-old female who presented with dyspnea only with no B symptoms and found to have left-sided chylothorax, and was later diagnosed to have stage IV follicular lymphoma. This case highlights an atypical presentation of follicular lymphoma, to help physicians to reach diagnosis earlier in similar cases.

## 1. Introduction

A chylothorax is an uncommon cause of pleural effusion [1]. It is caused by obstruction or injury to the thoracic duct or its tributaries, or transdiaphragmatic flow from peritoneal cavity. It often has a milky appearance with pleural fluid showing elevated triglycerides >110 mg/dL or presence of chylomicrons. Chylothorax can be caused by traumatic causes in around 50% of cases, which could be surgical such as esophagectomy or corrective procedures for congenital heart disease, or nonsurgical such as blunt trauma, childbirth, or even sneezing. Nontraumatic causes include malignancy, lymphatic disorders, SVC thrombosis, infections, and chylous ascites [1]. We present a case of woman in her 40 s who presented with shortness of breath and chylothorax as an initial presentation with no B symptoms and was later diagnosed to have follicular lymphoma.

## 2. Case Presentation

**Figure 1:**
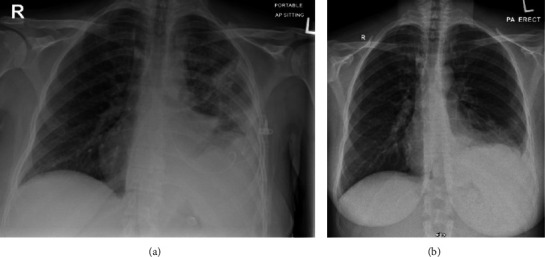
Chest XR (a) in the initial presentation showing left lower zone opacity obscuring left costophrenic angle suggestive of left-sided pleural effusion (b) after the chest tube drain is removed.

A woman in her 40 s with no past medical history presented with 1 week history of dyspnea that was on minimal exertion then progressed to rest. She did not have any fever, night sweats, or weight loss. She did not feel any new lymph nodes in her body, including in her breasts. She looked well in appearance and was not in any acute distress. Her vital signs were unremarkable, saturating well on room air. Physical examination revealed a small <1 mm axillary lymph node. On chest examination, there was decreased breath sound with dullness to percussion in the left lower zone of the chest. No hepatomegaly was felt on abdominal exam. Chest XR (Figure 1(a)) revealed large left-sided pleural effusion. Therapeutic thoracocentesis drained 1.6 L of milky pleural fluid, and fluid analysis showed exudative fluid with a protein ratio of 0.608 with lymphocytic predominance with 94%, high triglycerides 13.9 mmol/L (250.2 mg/dL), and highly suggestive of chylothorax as >110 mg/dL. Serum LDH was 248 U/L (135–214 I/U); however, pleural LDH was hemolyzed despite sending several samples. Cytology from pleural fluid showed no atypical or malignant cells. Repeated chest XR revealed almost complete resolution of left-sided pleural effusion (Figure 1(b)). Computed tomography of the neck, thorax, abdomen, and pelvis was done as malignancy was one of the differentials for lymphocytic pleural effusion, and it showed active nodal disease above and below the diaphragm, with large retroperitoneal mass. The retroperitoneal mass core biopsy revealed follicular lymphoma, grade 1-2 (follicular 30% and diffuse 70% architecture). There was re-accumulation of fluid and 1.7 L of chylous pleural fluid was drained by thoracocentesis. Positron emission tomography scan (PET scan) for staging showed metabolically active disease above and below the diaphragm with skeletal involvement and retroperitoneal confluent soft tissue. SUV levels in involved lymph nodes were ranging between 8.2 and 10.2, left-sided pleural effusion was nonavid, and left lower lobe consolidation was mildly avid. She was diagnosed with stage IVE G2 follicular lymphoma and transferred to specialized cancer center to be started on chemotherapy regimen rituximab, cyclophosphamide, doxorubicin hydrochloride, vincristine sulfate, prednisone (R-CHOP). At four months follow up, she had completed 4 cycles of chemotherapy with complete metabolic remission, and with no re-accumulation of pleural fluid.

## 3. Discussion and Conclusion

The most common presentation of follicular lymphoma is painless lymphadenopathy, which tends to have an indolent course [2]. Systemic symptoms such as night sweats, fever, and weight loss occurs in around 20% of cases [3]. Chylothorax, if it occurs, usually occurs late in the disease course [3]. It is rare to have an initial presentation as chylothorax, with 6 case reports of such cases in literature [3–9].

Different databases including PubMed and Google Scholar were searched for articles in English language with key words of “follicular lymphoma,” “non-Hodgkin lymphoma,” and “chylothorax,” which yielded 6 case reports with chylothorax as initial presentation of follicular lymphoma. The reported cases were stage IV at presentation, suggesting that chylothorax occurs late in the disease (Table 1). Four out of six cases presented with dyspnea without any B symptoms like our case (Table 1). The side of effusion was equal between left and right, with two cases having bilateral effusion (Table 1). Pleural fluid analysis for these cases showed exudative effusion, lymphocytic predominance, and high triglycerides levels far exceeding diagnostic criteria of chylothorax of >110 mg/dL, as in our case (Table 1). Three out of six cases as well as our case had recurrent accumulation of chylous effusion requiring either repeated therapeutic thoracocentesis or thoracic duct clipping (Table 1). The chylous effusion resolved and did not recur after starting on chemotherapy, implying the importance of addressing the underlying cause.

Sensitivity of pleural fluid cytology has been reported to be around 50–60% [10], and in our case, the cytology was negative. Although there will be differences between hospitals, it took 3 days for cytology to report in our hospital, making it feasible to wait for cytology prior to arranging for further tests such as biopsy to decrease unnecessary interventions.

Currently, there are no evidence-based guidelines for management of chylothorax [1]. Chylothorax is usually treated by pleural fluid drainage via indwelling catheter. Chylothorax is made up of mainly triglycerides from lymphatic system. As long-chain triglycerides travel in lymphatic system and medium-chain triglycerides get absorbed in portal vein and bypass the lymphatic system, avoiding long-chain triglycerides and instead using medium-chain triglycerides lead to decreased lymphatic flow and subsequently decreased chylous pleural fluid accumulation. Thus, dietary modification can be used, with high protein, low-fat diet (<10 g/day fat).

Somatostatin analogues such as somatostatin and octreotide, reduce chyle production, lymph flow, and intestinal fat absorption. It can be used with dietary modification to reduce accumulation of chylous pleural effusion.

If medical therapy fails, surgical intervention such as thoracic duct ligation, thoracic duct embolization, or medical or surgical pleurodesis can be done [1].

Chylothorax can be a rare initial presentation of a type of non-Hodgkin lymphoma, follicular lymphoma. Treatment of chylothorax should focus on pleural fluid drainage for diagnosis and symptomatic treatment and treating the underlying disease, such as chemotherapy. Also, dietary modification and somatostatin analogues, as well as surgical intervention if medical intervention fails, can be considered.

## Figures and Tables

**Table 1:** Literature review of case reports of initial presentation with chylothorax for follicular lymphoma.

Case	Reported year	Author	Cancer stage	Age	Gender	Pleural fluid analysis	Amount of pleural fluid drained	Presence of B symptoms	Effusion site	Chemotherapy	Treatment for chylothorax	Outcome
1	2015	Sidhertha	IV	75	Male	Exudative, 88% lymphocytes, cholesterol 96 mg/dL, triglyceride 826 mg/dL	1.6 L	Weight loss	Bilateral	R-CHOP	Drainage	Unknown
2	2018	Sugeeth	IV	47	Female	87% lymphocytes, cholesterol 50 mg/dL, triglyceride 256 mg/dL	Unknown	Weight loss	Left	R-CHOP	Drainage	Complete remission for 18 months
3	2021	Senem	IV	31	Male	Exudative, high triglyceride	Unknown	None	Left	R-CHOP	Drainage, follow up for persistent chylothorax	Significant regression in lymphadenopathy
4	2021	Akhilesh	IV	62	Female	Exudative, cholesterol 137 mg/dL, triglyceride 965 mg/dL	1.6 L	None	Bilateral + ascites	Unknown	Drainage, dietary modification	Unknown
5	2023	Andra	III	73	Female	Exudative, 97% lymphocytes, triglyceride 299.16 mg/dL	1.5 L	None	Right	R-CVP	Drainage, thoracic duct clipping, TPN, dietary modification	Complete remission
6	2023	Kyle	IV	64	Female	Exudative, 96% lymphocytes, cholesterol 69 mg/dL, triglyceride 747 mg/dL	1.5 L	None	Right	R-CHOP	Drainage	Recurrence of chylothorax requiring catheter, remission for 6 months

## Data Availability

The data for this case report are located at Hamad General Hospital, Doha, Qatar.
